# Laterality Does Not Affect the Depth Perception, but Interpupillary Distance

**DOI:** 10.1155/2013/485059

**Published:** 2013-11-27

**Authors:** Murat Aslankurt, Lokman Aslan, Adnan Aksoy, Murat Özdemir, Şenol Dane

**Affiliations:** ^1^Department of Ophthalmology, Faculty of Medicine, KSU, 46050 Kahramanmaras, Turkey; ^2^Department of Physiology, Faculty of Medicine, Turgut Özal University, 06170 Ankara, Turkey

## Abstract

In this study, which investigates the relationship between the levels of stereopsis with eye and hand dominance or interpupillary distance, 120 healthy young volunteers were investigated. Eye dominance was determined by modified Miles technique following a complete eye examination. Handedness was assessed with the Edinburgh handedness inventory. Interpupillary distance was measured with millimetric ruler. Stereoacuity was measured in both contour (Titmus test) and random dot (TNO test) stereograms. The stereopsis scores were evaluated in terms of hand or eye dominance. The correlation between stereopsis score and interpupillary distance was assessed. Main outcome measures were stereopsis scores according to hand and eye dominance. As a result, right- and left-handed individuals showed no differences in terms of stereopsis. No differences were found in stereopsis scores between right- and left-eye dominant people. There was a correlation between interpupillary distance and the depth of stereopsis (*r* = −0.248, *P* < 0.05). Contrary to the expectation, the left and right dominant individuals did not differ in levels of stereopsis. Interpupillary distance has a positive effect on stereopsis.

## 1. Introduction

In humans, while the right hemisphere is dominant for visuospatial functions like architecture, geometry, and mathematics, the left hemisphere is dominant for verbal functions like speech, literature, and poetry [1, 2]. A previous study found that left-handers evaluate overall perceptual similarity faster than right-handers, and they also mentally rotate perceived patterns of discrepant orientations faster than right-handers [3]. Additionally, left-handers have been found to have better performance in detecting rotations of three-dimensional examples [4, 5]. Based on these previous findings, we want to establish whether three-dimensional visual acuity is in any way related to eye and hand dominance.

There is an asymmetry in the use of eyes. This asymmetry is defined in different terms and determined by various tests. One of these terms, eye dominance is determined by the alignment of two objects presented at a stereodisparity far beyond Panum's area [6]. Miles test is one of the sighting tests [7]. There are reports in previous studies suggesting that when the dominant eye was closed in the Miles test, the shifting distance of the far point from focusing point in the horizontal plane was not similar for all subjects [7, 8]. In another study, the distance of focusing points of two eyes in the horizontal plane was greater in the right-handers than in the left-handers [9]. Another aim of the present study is to address the question of whether the shifting distance of the far point is a result of eye dominance or interpupillary distance.

## 2. Material and Methods

Informed consent was obtained from each participant. The study was approved by the local University Ethics Committee (2012/14-01) and conducted in accordance with the ethical principles described by the Declaration of Helsinki.

One hundred and twenty healthy young adult volunteers consisting of 58 men and 62 women, of mean age 26.11 ± 6.58 years (range: 18–56), were recruited. Subjects with strabismus, anisometropia, amblyopia, eyes without 20/20 vision, and a mental capacity that cannot handle the tests were excluded from the study. Subjects with more than one diopter of spherical equivalent refractive error were also excluded from the study.

Hand preference was determined using the Edinburg Handedness Inventory [10]. Subjects with handedness scores less than zero were considered as left-handed, subjects with scores greater than zero were considered as right-handed.

Ocular dominance was determined by using the near-far alignment test (Miles test) under after at least one-week correction of refractive errors, if needed [7]. First, two near and far points were identified: the near point was the tip of a stick 40 cm away from a constant jaw support, and the far point was marked on a wall 3 meters away from the first point. Vertical lines were drawn with 5 cm intervals on both sides of this point in the horizontal plane. The subject's jaw was fixed on the jaw support, and the subject was asked to focus both eyes on the far point on the wall using the tip of the stick as a reference point. Then, the subject was asked to close one eye without moving his head and eyes. The same procedure was repeated for the other eye. Shifting amount was inquired and noted in each case. If the tip of stick shifted from the far point in the horizontal plane when one of the eyes was closed, then the “closed” eye was considered to be the dominant eye.

Interpupillary distance was measured with a millimetric ruler while the subject looked at a fixation point 66 cm away.

Stereoacuity was measured in the sitting position at 40 cm in both contour (Titmus Fly test) and random dot (TNO test) stereograms under photopic conditions (85 cd/m^2^) with the presbyopia correction, if needed. The tests were performed through polarizing spectacles. The test plates were held at a 45° angle to the facial plane. Readings were recorded as arc per second (arc/s).

Chi-square test was used to compare categorical data, and *t*-test for quantitative data. Pearson's test was used to identify any potential quantitative correlations between the data, whereas Spearman's test was used for categorical correlations. Probability values < 0.05 were accepted to be significant. SPSS 17.0 was used for analysis purposes.

## 3. Results

The distribution of ages in men and women was similar. 94 subjects (78.3%) were right-handed and 26 (21.7%) were left-handed. Miles test showed that 92 (76.7%) participants were right-eye dominant, and 28 (23.3%) participants were left-eye dominant (Table 1). Out of the 92 right-eye dominant individuals, 78 (84.8%) were right-handed and 14 (15.2%) were left-handed. Left-eye dominant individuals had higher left handedness (46.2%). Eye and hand dominance were correlated (rho = 0.284, *P* < 0.01).

Right- and left-handed individuals did not show any difference in terms of stereopsis. No differences were found between the right- and left-eye dominant people in terms of stereopsis scores (Tables 2 and 3).

**Figure 1:**
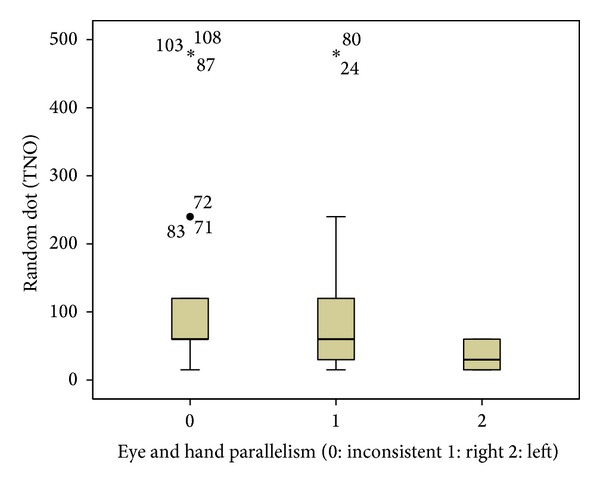
Stereopsis scores in TNO test according to the eye and hand parallelism (*P* < 0.01, One-way ANOVA). Left-sided people have better stereopsis scores than right sided and inconsistent ones.

Hand and eye dominance consistencies were evaluated. If hand and eye dominance were parallel, it was called left or right “sided,” otherwise “inconsistent.” Stereopsis scores of the left-sided people were better than right-sided or inconsistent people (ANOVA *P* < 0.01) (Figure 1).

An evaluation of the whole male and female group showed a mean interpupillary distance measurement of 62.5 ± 3.9 mm. Female participants had significantly lower interpupillary distances than men (61.4 ± 3.4, 64.4 ± 4.0 mm, *P* < 0.01). Although interpupillary distance was lower in women than in men (*P* < 0.01), stereopsis scores were better. However, the difference was not statistically significant (*P* = 0.085 for TNO and *P* = 0.092 for Titmus Fly) (Table 4).

**Figure 2:**
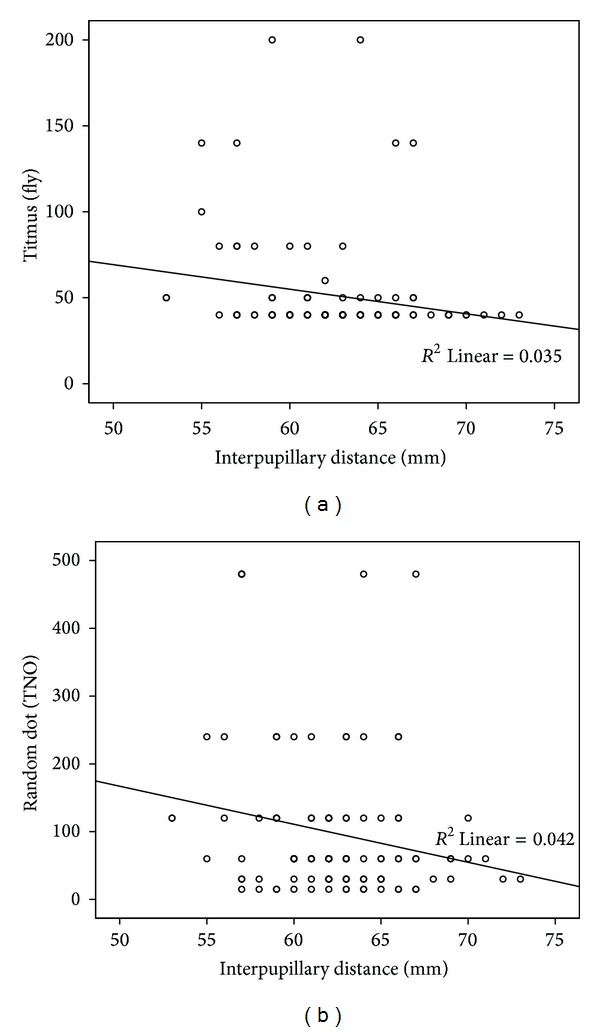
Correlation with interpupillary distance and stereopsis scores in Titmus and TNO tests. Stereopsis scores are increased with interpupillary distance.

Age and interpupillary distance showed a positive correlation as expected (*r* = 0.337, *P* < 0.01). There was a significant but weak negative correlation between interpupillary distance and stereopsis scores in both Titmus and TNO tests (*r* = −0.248, *P* < 0.01, *r* = −0.167, *P* < 0.05, resp.) (Figures 2(a) and 2(b)).

**Figure 3:**
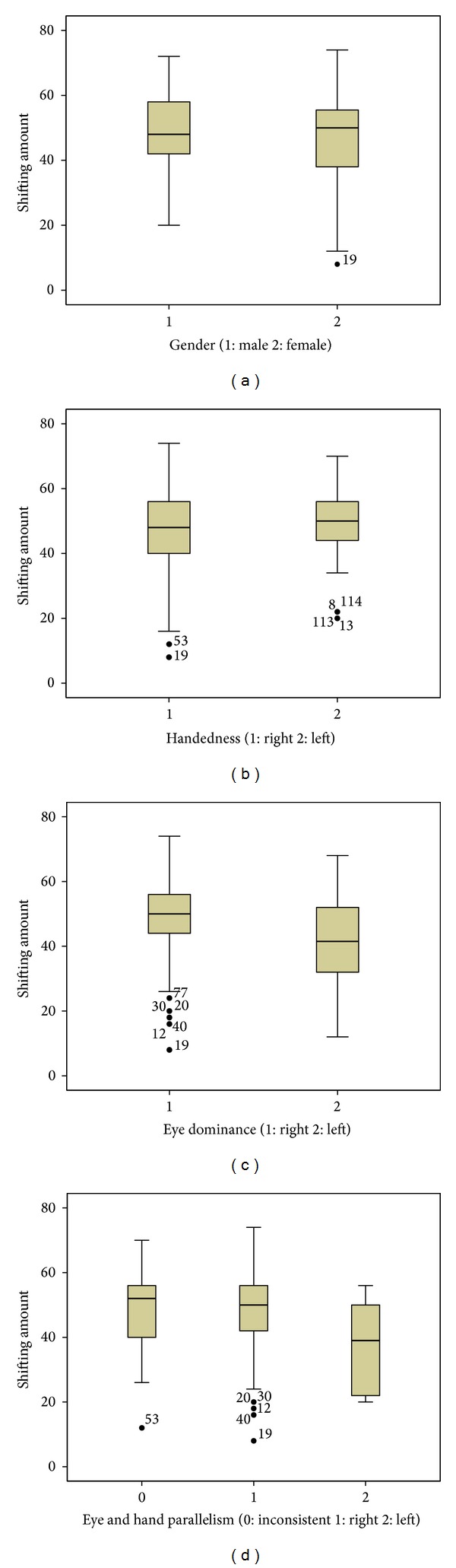
Box plots of shifting amount of far points in Miles test. (a) Gender, (b) handedness, (c) eye dominance, and (d) eye and hand parallelism. Right eye dominant subjects showed greater amount of shifting in Miles test.

Average stereopsis scores of the group were better in Titmus test (56.0 ± 36.6) than in TNO test (98.1 ± 102.4). However, individual stereopsis scores as measured by two methods showed a significant correlation between each other (*r* = −0.637, *P* < 0.01). Shifting distance from far point in Miles test was greater in the right-eye dominant subjects than inthe left-eye dominants (48.7 ± 13.5 and 41.2 ± 14.1, resp., *P* < 0.05). However, they were equal in right- and left-handers (*P* > 0.05) (Figure 3).

**Figure 4:**
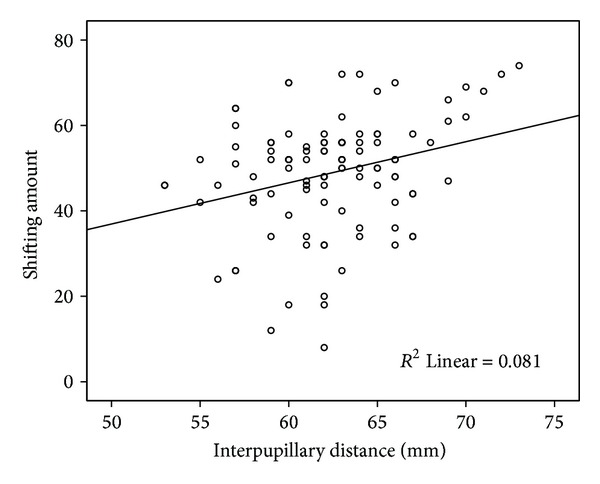
There is a positive correlation with interpupillary distance and shifting in Miles test.

Shifting distance and the degree of stereopsis were not correlated. Interpupillary distance and shifting amount from far point were correlated to each other (*r* = 0.358, *P* < 0.01) (Figure 4).

## 4. Discussion

In addition to being a result of the optical properties of both eyes, stereopsis is a complex process on the participation of the cerebral cortex [11, 12]. A study shows that stereoacuity was affected by right cerebral disease, but not left cerebral disease [13]. Left-handers are faster in same-different judgment of visual patterns than right-handers [3]. They are also better in detecting the rotations of three-dimensional examples [4, 5]. Right or left side dominance differs in perceptions, senses, and skills [14]. Therefore, this creates an expectation that stereopsis can also differ. However, contrary to the expectation, hand or eye dominance was not found to correlate with the level of stereopsis in the present series. Stereopsis scores were remarkably close in right- and left-handed participants, but the stereopsis level in the right-eye dominant subjects was slightly greater than that in the left-eye dominant. Ocular prevalence and stereoacuity and stereoscopic prevalence and binocular functions were mentioned in the literature [15, 16]. However, to the best of our knowledge, this is the first study addressing relationships between stereopsis and laterality.

The Titmus and Random dot tests are the most common tests for stereopsis measurement, and are valued clinically because they are compact, easy to store and carry, and quick to administer and score. Titmus test can analyze local stereoacuity with large ranges from 800 to 40 sec arc in nine steps while TNO analyzes 400 to 20 sec arc [17]. In the present series, stereopsis scores in TNO were worse than in Titmus. This can be related to the random dot stereograms being relatively more difficult than contour stereograms. However, individual stereopsis scores obtained from TNO and Titmus tests were correlated with each other.

The third dimension becomes noticeable in a single scene by two images coming from two eyes looking at an object at a slightly different perspective. The increased depth of stereopsis with a longer interpupillary distance may be a consequence of the wide variation of images obtained from the object. Although interpupillary distance was smaller in women than in man, stereopsis levels were similar, even slightly better. This implies that stereoacuity is a multifactorial process.

The amount of shift in far point in Miles test was greater in right eyes in left-handers than in the right-handers and was greater in left eyes in right-handers than in the left-handers [9]. Similar results were obtained in the present series but were not found to be statistically significant. Dane and Gümüştekin also found a negatively significant correlation between hand preference and the distance of focusing points of right eyes, with a positive correlation between hand preference and distance focusing points of left eyes. Hence, it was postulated that hand preference may be related to the degree of ocular asymmetry [9]. Although shifting amount was reported bigger in the right eye dominant subjects there were no correlations between hand preference and shifting points of eyes in the present series. On the contrary, our series showed a correlation between interpupillary distance and the amount of shift.

As a result, the left or right dominance appears to have no effects on binocularities. However, interpupillary distance makes a positive contribution to stereopsis. The amount of shift in far point in Miles test may be a result of the interpupillary distance.

## Figures and Tables

**Table 1:** The distribution of gender and eye and hand dominance.

Dominance	Side	Male (*n*, %)	Female (*n*, %)	Total (*n*, %)
Hand	Right	45 (77.5)	49 (79)	94 (78.3)
	Left	13 (22.5)	13 (21)	26 (21.7)

Eye	Right	45 (77.5)	47 (75.8)	92 (76.7)
	Left	13 (22.5)	15 (24.2)	28 (23.3)

**Table 2:** Stereopsis scores and shifting amount in Miles test according to eye dominance.

	Right	Left	Probability
TNO	97.42 ± 104.17	97.50 ± 108.43	0.99
Titmus	53.86 ± 31.65	63.57 ± 52.08	0.23
Shifting	49.22 ± 13.29	40.43 ± 14.09	0.003

**Table 3:** Stereopsis scores and shifting amount in Miles test according to hand dominance.

	Right	Left	Probability
TNO	99.03 ± 99.05	91.25 ± 104.71	0.74
Titmus	55.60 ± 35.77	56.92 ± 43.52	0.90
Shifting	47.11 ± 13.87	47.38 ± 14.41	0.92

**Table 4:** Interpupillary distance and stereopsis scores of each gender.

	Male	Female	Probability
IPD	64.38 ± 38	61.32 ± 3.40	*P* < 0.001
Titmus	63.56 ± 44.62	51.67 ± 31.79	*P* = 0.092
TNO	119.30 ± 124.44	84.73 ± 81.01	*P* = 0.085
